# Supplementary material for: Mild Paravalvular Leak May Pose an Increased Thrombogenic Risk in Transcatheter Aortic Valve Replacement (TAVR) Patients-Insights from Patient Specific In Vitro and In Silico Studies
Source: Bioengineering (Basel). 2023 Feb 1;10(2):188. doi: 10.3390/bioengineering10020188 (PMC9952825; doi:10.3390/bioengineering10020188)
Supplement: Supplementary file 1 [file bioengineering-10-00188-s001.zip › bioengineering-2164464-supplementary.pdf]

Supplementary Materials

Mild paravalvular leak may pose an increased thrombogenic risk in transcatheter aortic valve replacement (TAVR) patients - Insights from patient specific *in vitro* and *in silico* studies

Short title: Thrombogenic Risk of Mild Paravalvular Leak in TAVR

Brandon J Kovarovic[1], Oren M Rotman PhD[1], Puja Parikh MD[2], Marvin Slepian[3], Danny Bluestein PhD[1]

[1] Biofluids Research Group, Department of Biomedical Engineering, Stony Brook University, Stony Brook, NY, USA.

[2] Division of Cardiovascular Medicine, Department of Medicine, Stony Brook University, Stony Brook, NY, USA.

[3] Sarver Heart Center, University of Arizona, Tucson, AZ, USA

Correspondence:

Professor Danny Bluestein PhD

Department of Biomedical Engineering, Stony Brook University

T8-050 Health Sciences Center

Stony Brook, NY 11794-8084, USA

Tel: +1 (631) 444-2156

Email: danny.bluestein@stonybrook.edu

IRB approval: 2013-2357-R5, 2/10/2020

Additional Images of platelet velocity and stresses – Cartesian projections

Supplementary Figures S1 and S2 feature 3 cartesian angles of the anatomies A-E with platelet pathlines over the 3 cardiac cycles. Platelets are colored by velocity and stress magnitude (defined by Equation 2). These figures help provide a complete image of each anatomy where the polar projections in Figure S5 can be abstract to easily follow.

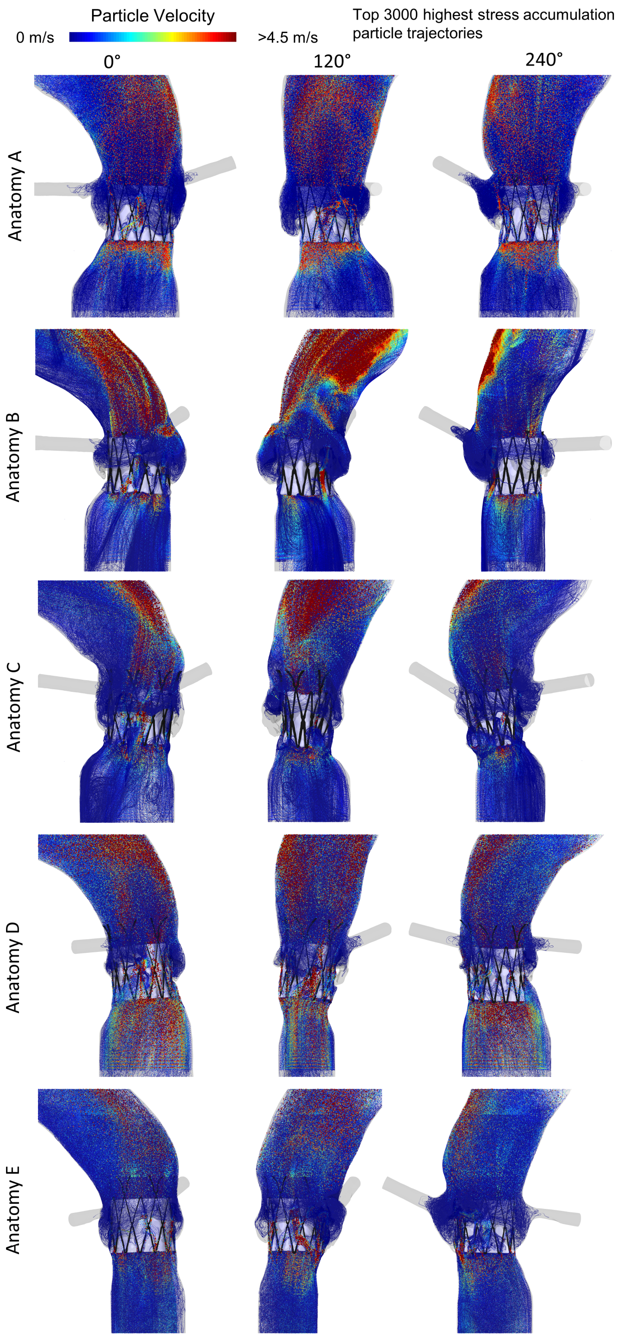

Supplementary Figure S1: Three views of top 3000 highest SA platelet pathlines colored by velocity.

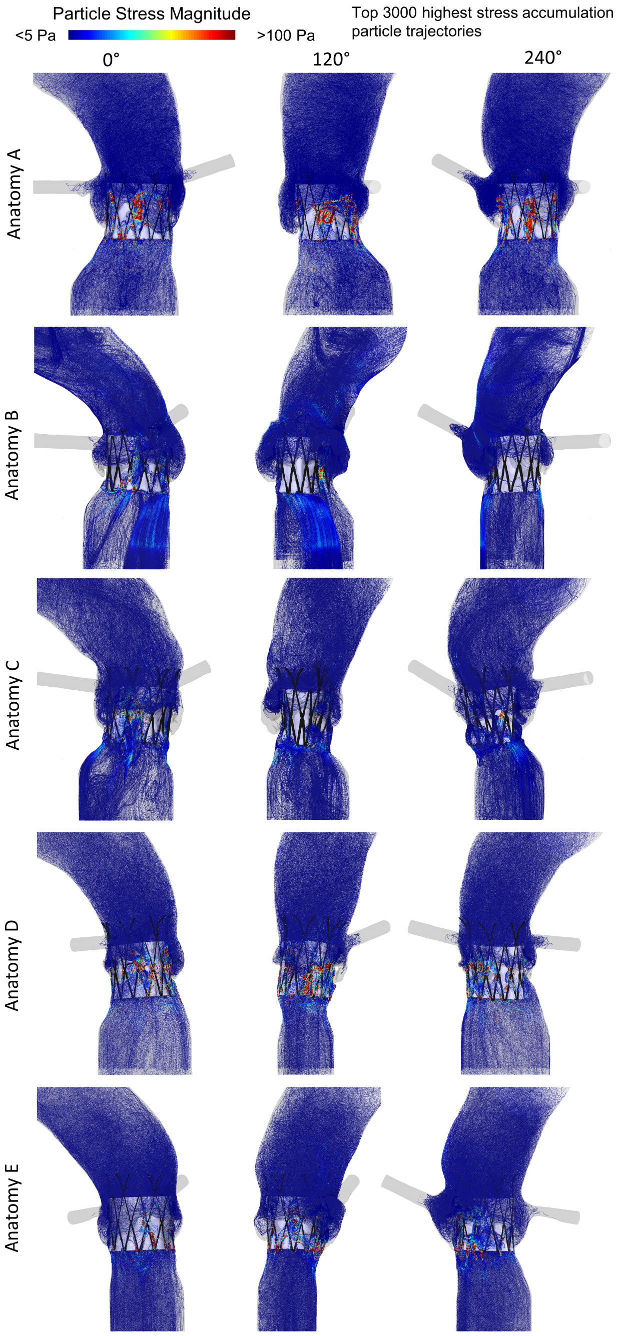

Supplementary Figure S2: Three views of top 3000 highest SA platelet pathlines colored by stress magnitude.

Additional Images of diastolic streamline analysis – Polar and cartesian projections

Supplementary Figures S3–S5 feature polar projection and cartesian angles of the anatomies A-E with diastolic streamlines at peak diastole (single timestep). The streamlines are colored by velocity and distance to the wall which as used to approximate the instantaneous Reynold's number. Streamlines were seeded 2 cm proximal to the TAVR commissures.

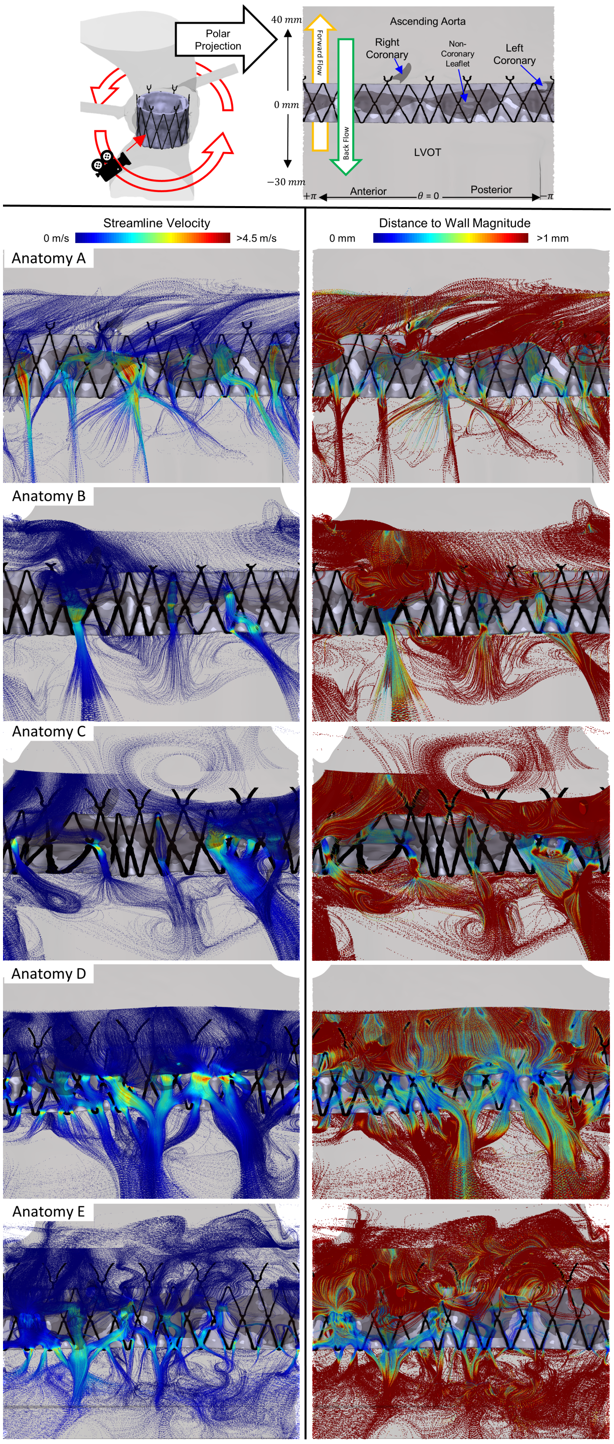

Supplementary Figure S3: Polar projections of the diastolic streamlines.

Top- key to visualize the polar projection. Left column is colored according to the streamline velocity magnitude and right column is colored by shortest distance to a wall boundary.

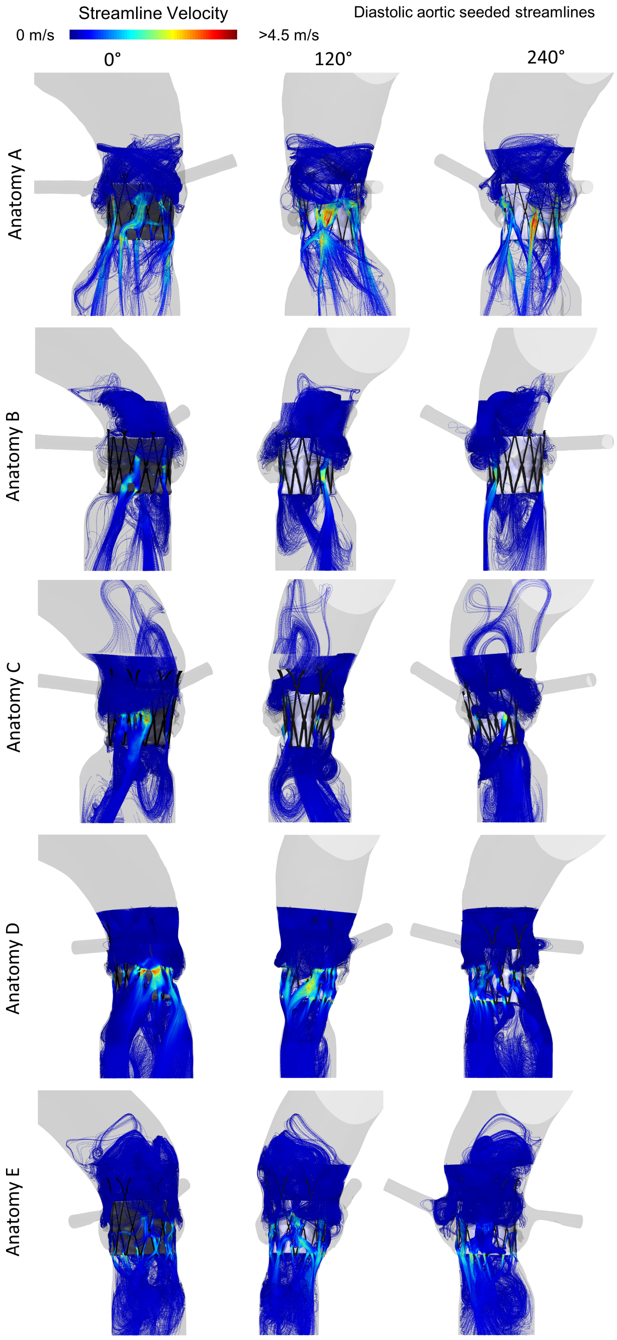

Supplementary Figure S4: Three views of the diastolic streamlines colored by velocity magnitude.

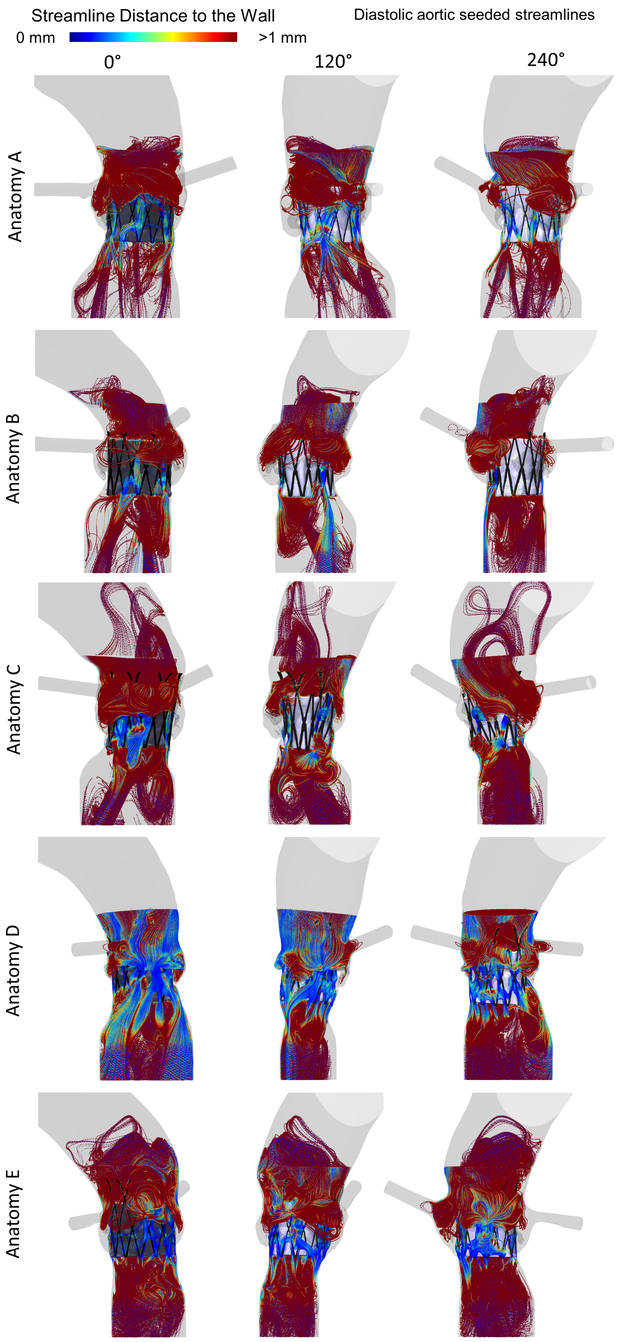

Supplementary Figure S5: Three views of the diastolic streamlines colored by shortest distance to a wall boundary.

Additional Images of example complex platelet trajectories

Supplementary Figure S6 demonstrates examples of complex platelet trajectories selected randomly from the platelets with multiple re-entrainments into the PVL flows. The trajectories enter and exit the PVL channels multiple times and in non-consecutive cycles.

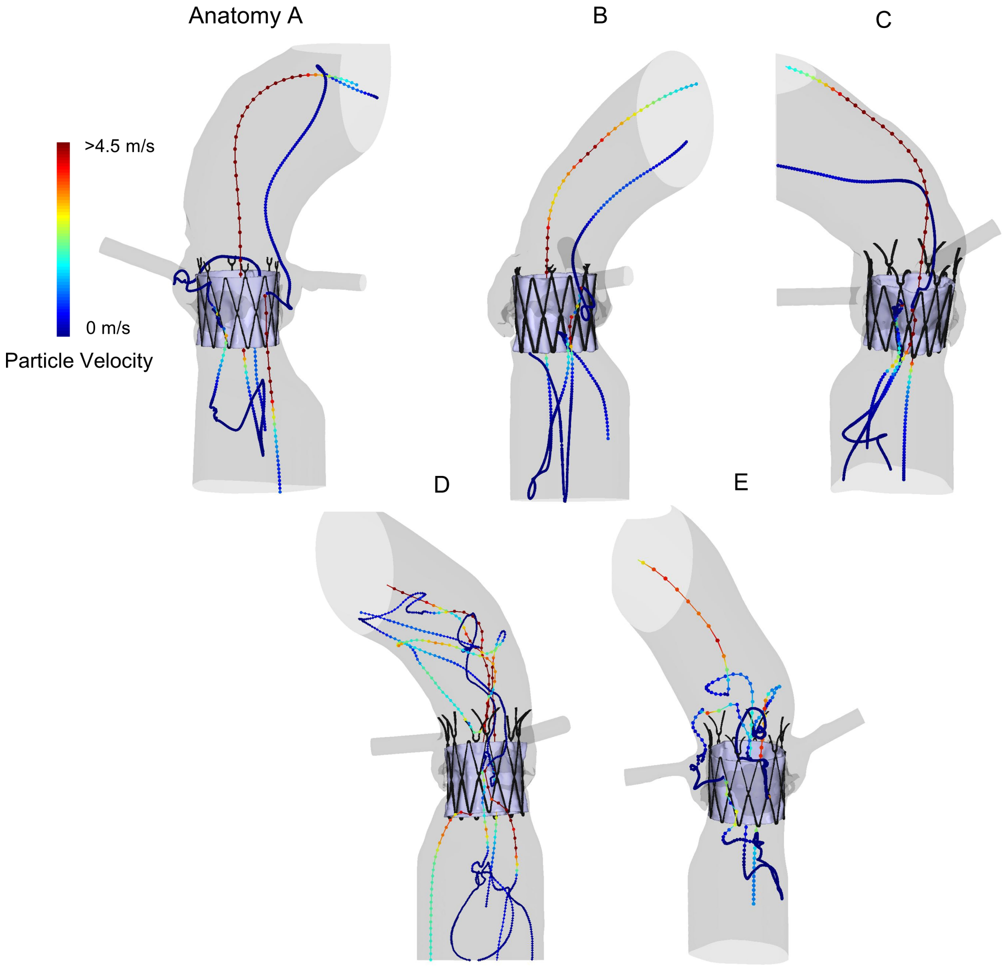

Supplementary Figure S6: Example complex platelet trajectories in each anatomy
Example platelet trajectories colored by velocity, randomly selected from platelets marked with multiple re-entry or re-entrainment into the PVL flows.

Analysis of platelet injection time versus impact on thrombogenic potential

The following figure is a comparison of three anatomies and platelet injection times. There is a difference between the single injection timepoint that was done with previous DTE studies and the in depth complete systolic injection completed in this study. More importantly, each anatomy demonstrates that the importance of injection time (early or late systole) varies the degree of thrombogenic potential. Therefore, it was concluded that in order to fairly compare each anatomy, the entire systolic period needed to be captured.

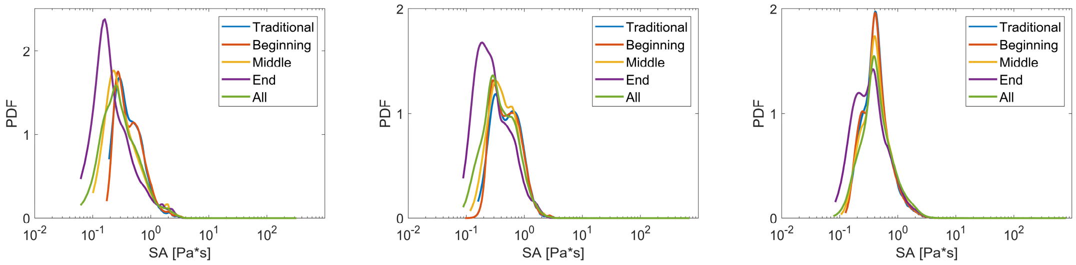

Supplementary Figure S7: PDF comparison versus injection time

Example PDF comparison of (left to right) Anatomy B, C, and D. "Traditional" curve (blue) represents an injection of platelets at a single plane at the beginning of systole that was conducted in previous DTE analysis of medical devices. "Beginning", "Middle", and "End" curves compile the each 1/3 of the systolic injections. "All" curve comprises all the platelets injected over the entire systolic period and tracked over 3 consecutive cycles.

Supplementary Table of linear correlations between hydrodynamic parameters and the thrombogenic indicators

Pearson correlation (r) coefficients between the simple hemodynamic in silico data and the DPM platelet results. Data normality cannot be confirmed with the limited (n=5) sample set and each data was needed to be further analyzed for validity.

			% of Platelet Entering PVL	% of Platelet Re-Entering PVL	Stress Accumulation of PVL platelets [Pa s]	
					Median	Mean
In Silico	CO [L/min]		-0.52	-0.43	0.61	0.46
	EOA [cm²]		-0.19	0.23	0.49	0.68
	EROA [cm²]		0.42	0.68	0.52	0.71
	Leak Flow [ml/beat]		-0.43	-0.56	-0.72	-0.84
	RF [% SV]		0.55	0.75	0.47	0.70
	% of Platelet Entering PVL		1.00	-	-	-
	% of Platelet Re-Entering PVL		0.89	1.00	-	-
	Stress Accumulation of PVL platelets [Pa s]	Median	-0.21	-0.17	1.00	-
		Mean	-0.07	0.08	0.94	1.00
		SD (σ)	0.01	0.18	0.87	0.98
Streamlines	Length [mm]	Mean	-0.11	-0.01	-0.26	-0.10
	Radii [mm]	Mean	-0.54	-0.43	-0.68	-0.68
	Velocity [m/s]	Mean	-0.44	-0.25	0.87	0.91
	Reynolds Number	Mean	-0.44	-0.21	0.77	0.85
		Max	-0.19	0.10	0.72	0.88
PVL Contour	Filtered PVL Velocity (>0.3 m/s) [m/s]	Mean	-0.56	-0.28	0.80	0.83
		SD (σ)	-0.15	0.30	0.29	0.53
		Max	-0.05	0.40	-0.04	0.23

Videos of platelet motion in each anatomy

Supplementary Video S1–S5 features rotating images and polar projections of the anatomies A-E with platelet locations over the final 3 cardiac cycles. Platelets are colored by velocity and stress magnitude (defined by Equation 2). In order better visualize the platelet paths, 1 million platelets were randomly selected.

Supplementary Video S1: Animation of 1 million platelets from Anatomy A colored by velocity and stress magnitude with polar projections.

Supplementary Video S2: Animation of 1 million platelets from Anatomy B colored by velocity and stress magnitude with polar projections.

Supplementary Video S3: Animation of 1 million platelets from Anatomy C colored by velocity and stress magnitude with polar projections.

Supplementary Video S4: Animation of 1 million platelets from Anatomy D colored by velocity and stress magnitude with polar projections.

Supplementary Video S5: Animation of 1 million platelets from Anatomy E colored by velocity and stress magnitude with polar projections.

The videos were uploaded following the instructions for larger file sizes to the Zenodo site. Please find them here: https://doi.org/10.5281/zenodo.7495020
