# Supplementary figures and images for: Mild Paravalvular Leak May Pose an Increased Thrombogenic Risk in Transcatheter Aortic Valve Replacement (TAVR) Patients-Insights from Patient Specific In Vitro and In Silico Studies
Source: Bioengineering (Basel). 2023 Feb 1;10(2):188. doi: 10.3390/bioengineering10020188 (PMC9952825; doi:10.3390/bioengineering10020188)

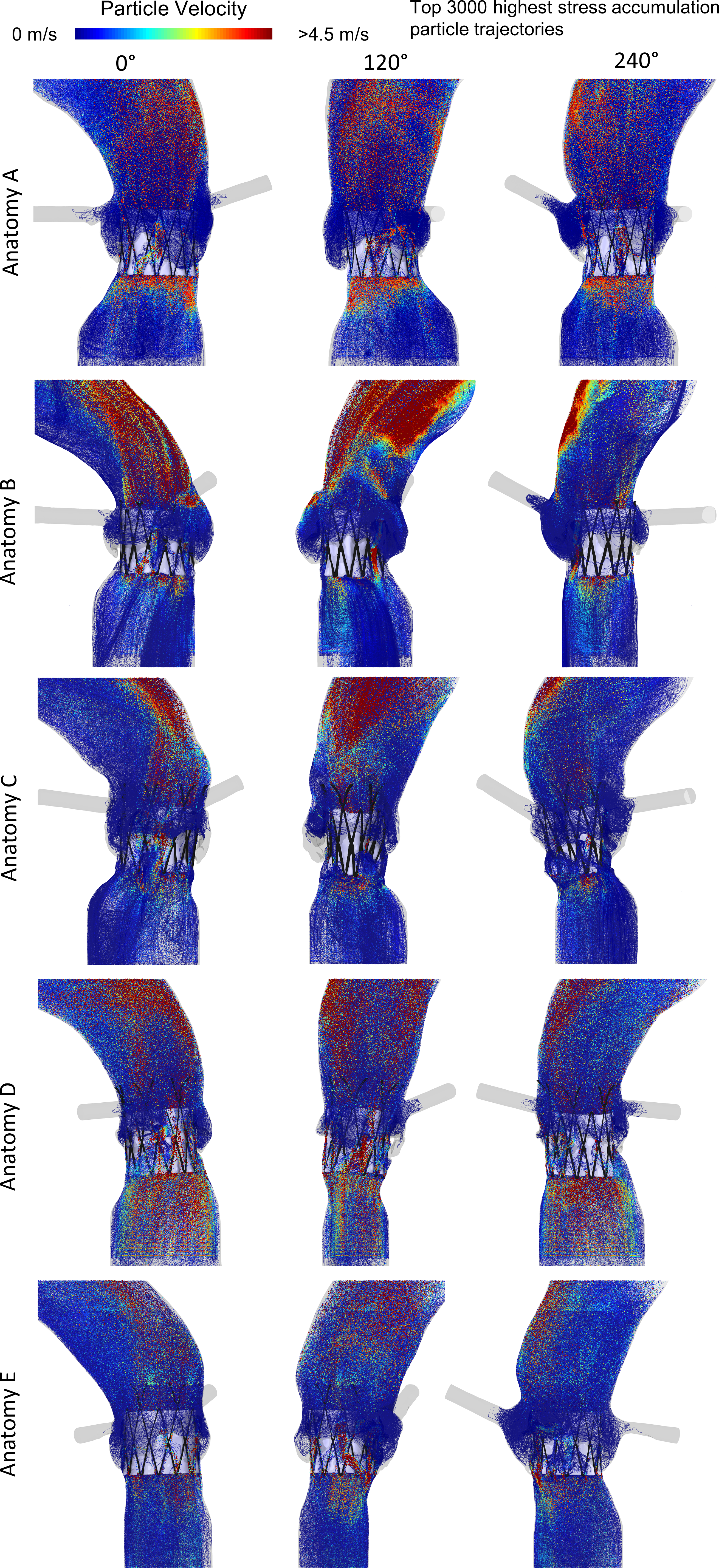

Supplement: Supplementary file 1 [file bioengineering-10-00188-s001.zip › Sup Fig 1.TIF]

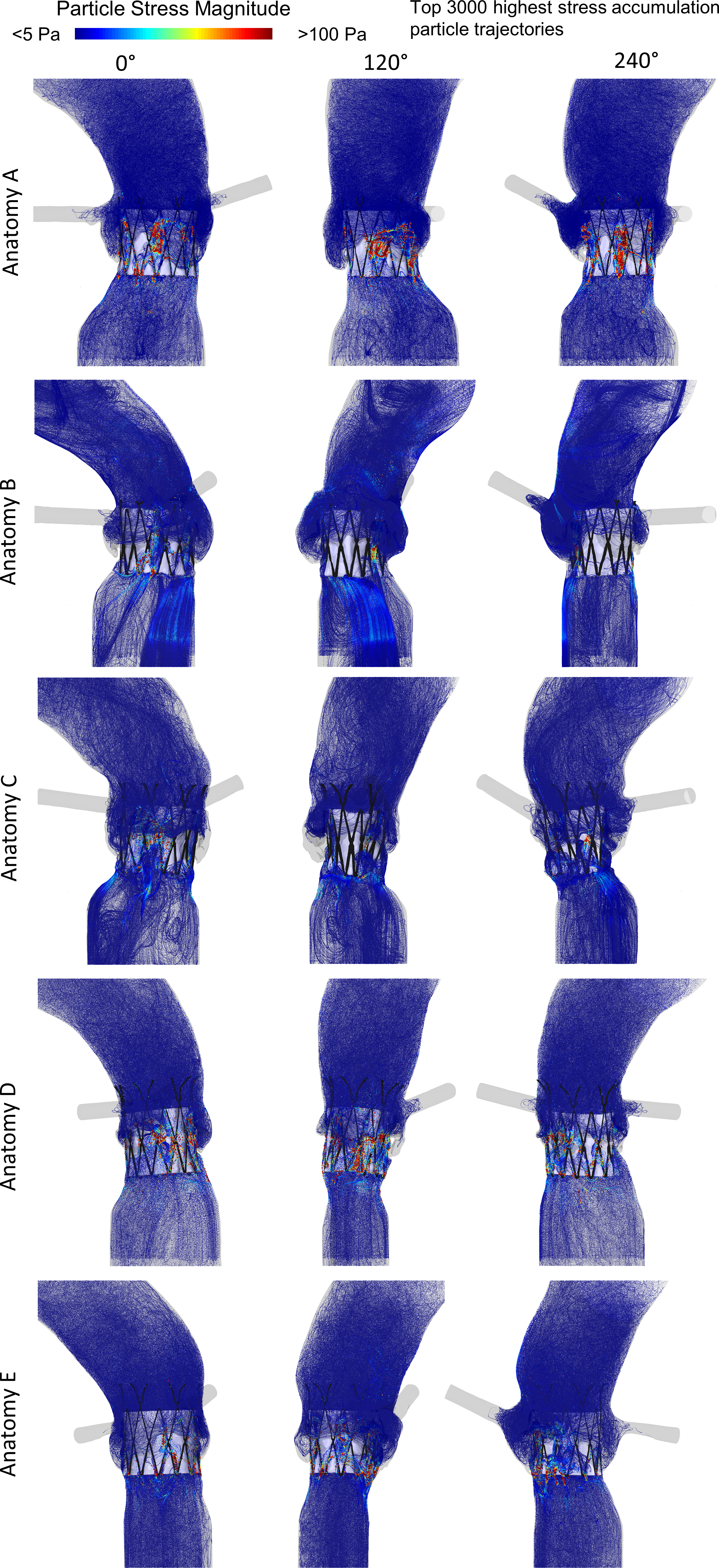

Supplement: Supplementary file 1 [file bioengineering-10-00188-s001.zip › Sup Fig 2.TIF]

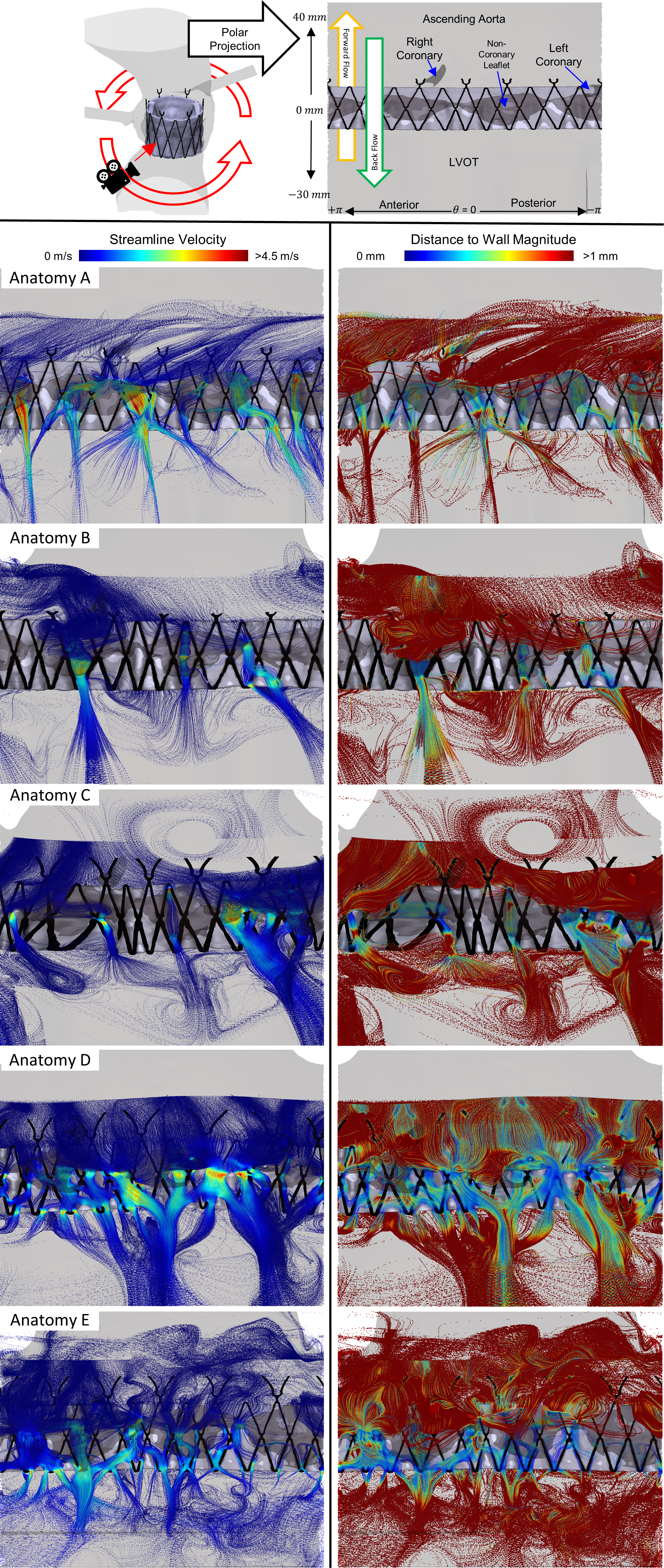

Supplement: Supplementary file 1 [file bioengineering-10-00188-s001.zip › Sup Fig 3.TIF]

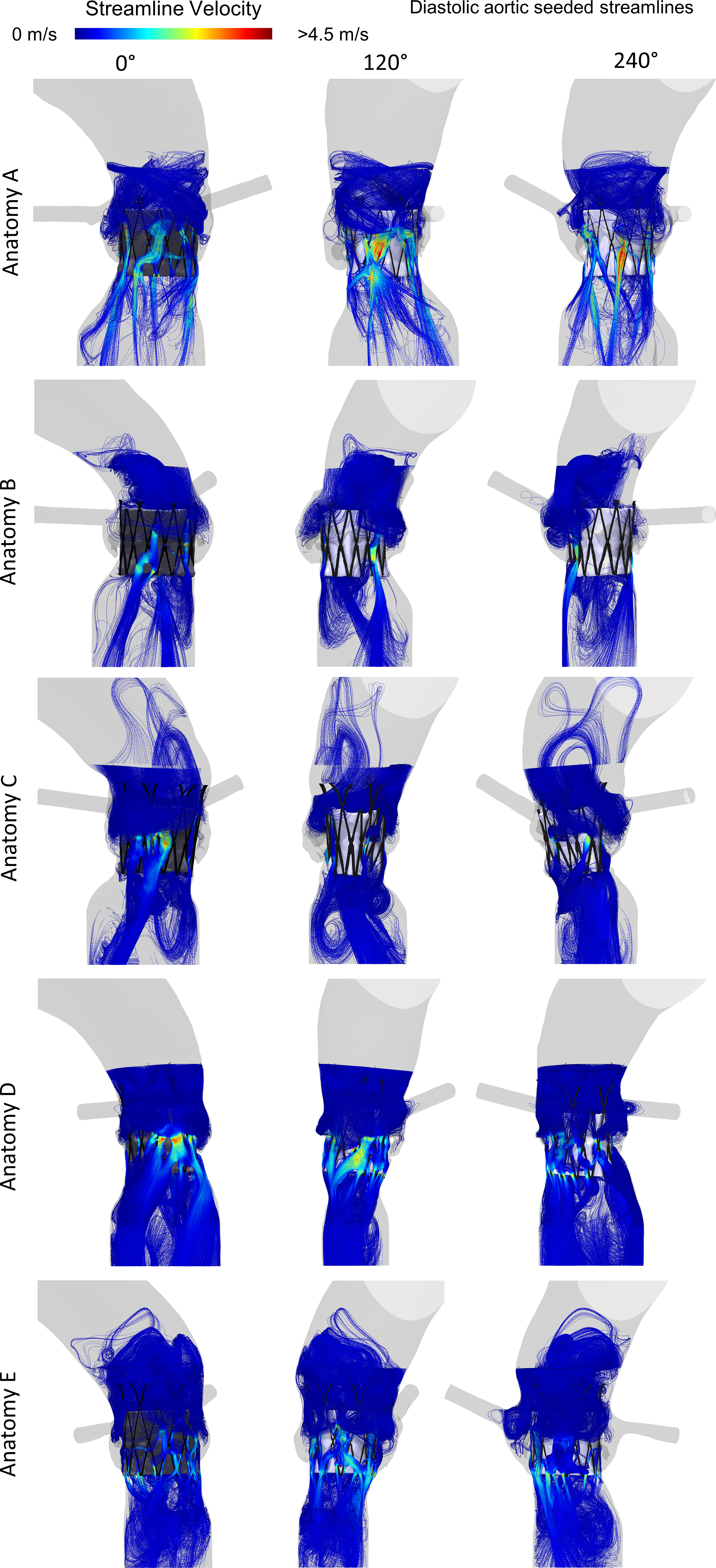

Supplement: Supplementary file 1 [file bioengineering-10-00188-s001.zip › Sup Fig 4.TIF]

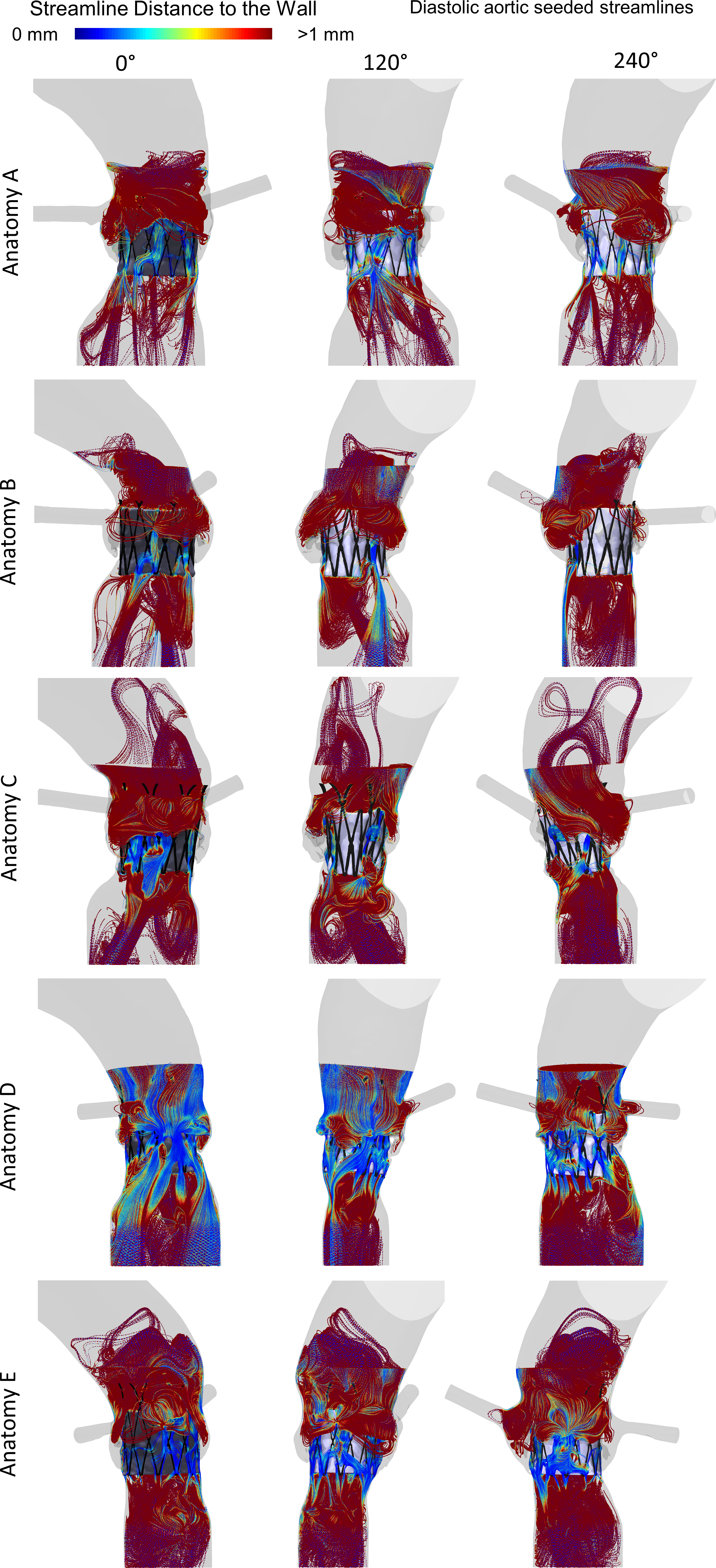

Supplement: Supplementary file 1 [file bioengineering-10-00188-s001.zip › Sup Fig 5.TIF]

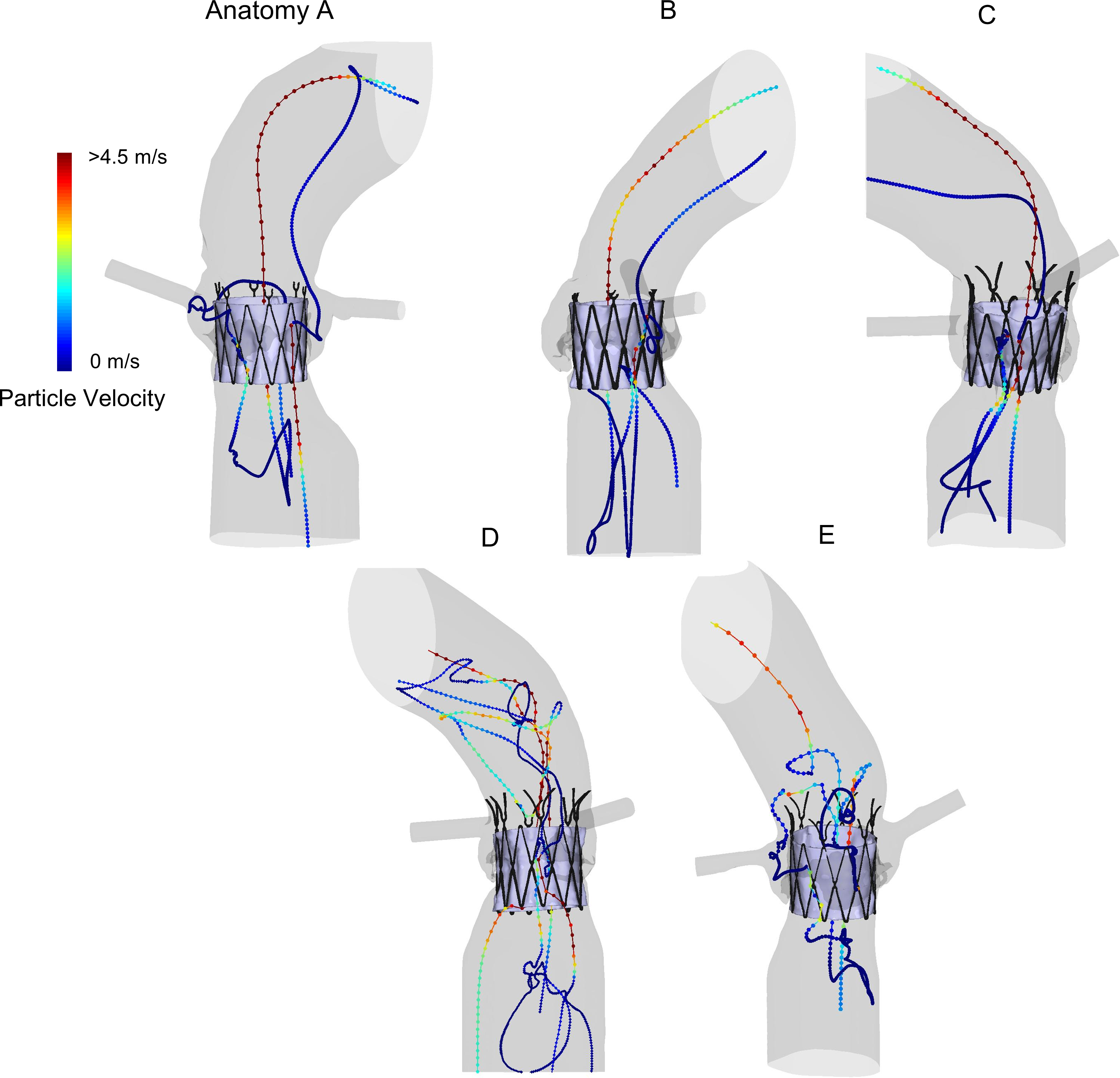

Supplement: Supplementary file 1 [file bioengineering-10-00188-s001.zip › Sup Fig 6.tif]
